# Inflammatory breast cancer: is it really a separate entity?

**DOI:** 10.3332/ecancer.2012.250

**Published:** 2012-04-12

**Authors:** AS Bastawisy, RM Gaafar, SS Eisa, GM Amira, MH Helal

**Affiliations:** 1 Medical Oncology, National Cancer Institute,Cairo University, Cairo, Egypt; 2 Pathology, National Cancer Institute,Cairo University, Cairo, Egypt; 3 Surgery, National Cancer Institute,Cairo University, Cairo, Egypt; 4 Radiology, National Cancer Institute,Cairo University, Cairo, Egypt

## Abstract

**Background:**

Inflammatory breast cancer (IBC) is the most aggressive form of primary breast carcinoma and is associated with a dismal outcome despite the availability of multi-modality treatment options.

**Patients and methods:**

This is a prospective case control study comparing two groups of newly diagnosed patients; the first with inflammatory breast cancer (IBC) and the second with locally advanced non inflammatory breast cancer (LABC). In both groups MIB1, ER, PR, Her2neu were assessed. Neo-adjuvant chemotherapy consisted of four cycles of FEC100 followed by modified radical mastectomy according to clinical response, postoperative chemotherapy with two courses of the same regimen followed by radiotherapy. Tamoxifen 20 mg po daily for 5 years in ER and/or PR positive tumours, starting after the completion of radiotherapy. Primary end points were a) comparison of MIB-1 score in both groups, b) comparison of clinical and pathological responses in both groups. Secondary endpoints were comparison of progression free survival and overall survival.

**Results:**

From a total of 42 patients, 21 were stage III B (T4d, N0-2 M0) IBC and 21 were stage III B (T4a-c, N0-2, M0) LABC. Patients in the age range from 28 to 68 were included and followed from November 2007 until February 2010 with a median follow-up period of 22.5 months. Toxicity of both arms, mainly haematologic, nausea and vomiting, was in general acceptable with no treatment-related deaths. Of the patients with IBC 81.3% had a high MIB-1 score as compared with 43.8% of patients with LABC (P-value = 0.028). Objective clinical response to neo-adjuvant chemotherapy in the IBC arm was 57.1% (4.8% complete response (CR)) as compared with 81% (9.5% CR) in LABC (P-value = 0.09). Overall pathological response (complete pathological response (pCR)+partial pathological response (pPR)) was 35.3% in the IBC arm compared with 40% in LABC arm (P-value = 0.618). One year, 2 year and median progression free survival (PFS) were 55.87%, 37.71% and 21.7 months, respectively in the IBC arm compared with 85.71%, 66.67% in LABC (median PFS was not reached) (P-value = 0.072). One and 2 year overall survival (OS) were 69.82% and 51.20%, respectively in the IBC arm compared with 95.24% and 95.24% in LABC arm (P-value = 0.0038).

**Conclusions:**

IBC should be considered as a separate entity. A high MIB-1 score is a potential molecular marker for IBC.

## Background

Inflammatory breast cancer (IBC) is an aggressive form of breast carcinoma accounting for 1–6% of all invasive breast tumours in the United States [1]. Although advances in aggressive multi-modality therapeutics have impacted survival in IBC, the prognosis remains poor with 3-year survival rates of 40% for IBC compared with 85% for non-inflammatory locally advanced patients with breast cancer [2]. Clinically, IBC is defined by a rapid onset of distinct features such as diffuse skin erythema, oedema involving more than two-thirds of the breast resulting in a pitted appearance (peau d’ orange), as well as tenderness, induration and warmth of the involved breast [1]. Pathologically, IBC frequently demonstrates dermal lymphatic invasion by tumour emboli however, absence of dermal lymphatic invasion does not exclude the diagnosis if the clinical signs are present [3]. In addition to the clinical and pathological characteristics described above, IBC had statistically higher proliferation as measured by Ki67 index (93% of IBC samples had a high proliferation index versus only 11% of non-IBC samples; P < 0.001 [4]). IBC tumours were more likely to be ER negative (49% versus 30%; P = 0.002) and PR negative (68% versus 42%; P = 0.001 [4]). The expression levels of signalling tyrosine kinases were not significantly different: EGFR was 23% and 19% while Her2 was 26% and 17% respectively in IBC and non-IBC samples [4]. RhoC has been reported to be overexpressed in 90 percent of IBC versus 38% of stage-matched, non-inflammatory breast cancers [5]. Combined-modality treatment (neo-adjuvant chemotherapy, mastectomy, adjuvant chemotherapy and radiotherapy) is considered to be a standard of care for IBC [6]. Pathologic complete response (pCR) in breast and axilla to induction chemotherapy has been identified as the strongest prognostic factor for locally advanced breast cancer, including IBC [7,8].

## Objectives of the study

The primary objectives of the study were to compare the efficacy of neo-adjuvant chemotherapy in inducing clinical and pathological response in inflammatory and non-inflammatory locally advanced breast cancer. We also wanted begin to establish a prognostic molecular phenotype for IBC including ER, PR and Her2 neu status and proliferative index as indicated by the level of MIB-1 antibody staining.

The secondary objective was to compare progression free and overall survival in inflammatory and non-inflammatory locally advanced breast cancer.

## Patients and Methods

This is a prospective case control study including all eligible cases of inflammatory breast cancer and an equal number of patients with non-inflammatory locally advanced breast cancer presenting to the National Cancer Institute (NCI), Cairo University during the period from November 2007 to November 2008.

### Inclusion criteria

Eligible cases of stage III breast cancer fulfilling the following criteria:

- Age not less than 18 years.
- Pathological confirmation of breast cancer.
- ECOG performance status (PS) 0–2.
- Adequate renal and hepatic functions.
- Ejection fraction more than or equal to 50%.
- No other malignancies.

Group (1): includes all cases of stage III inflammatory breast cancer which are diagnosed mainly based on clinical ground and supported by the presence of dermal lymphatic invasion by tumour emboli evidenced on pathological examination.

Group (2): includes eligible patients with non-IBC stage III breast cancer.

The study was conducted according to the Declaration of Helsinki and the guidelines for Good Clinical Practice. The local ethics committees approved the protocol, and informed consent was obtained from all patients before study entry.

### Evaluation

Initial evaluation of the patients included:

○ Complete history and physical examination.
○ CBC and chemistry.
○ Bilateral mammography and breast ultrasound
○ Chest x-ray.
○ Abdomino-pelvic ultrasound.
○ Echocardiography.
○ Bone scan.
○ Wedge biopsy including the skin for pathological examination.

Prior to each cycle:

○ Complete physical examination.
○ CBC and chemistry.

After four cycles:

○ Chest x-ray.
○ Abdomino-pelvic ultrasound.
○ Bilateral mammography and breast ultrasound.

### Treatment protocol

Both groups received four cycles of neo-adjuvant chemotherapy (FEC 100) regimen every 21 days as follows. Cyclophosphamide 500 mg/m^2^ IV diluted in 50 ml of normal saline as a 5- to 10-minute intravenous infusion on day 1; Epirubicin 100 mg/m^2^ IV diluted in 50 ml of normal saline as a 5- to 10-minute intravenous infusion on day 1; Fluorouracil 500 mg/m2 IV D1 as a bolus intravenous injection.

Modified radical mastectomy was performed after full assessment of response and restaging within 3 weeks from the last chemotherapy session in non-progressive cases; postoperative chemotherapy with two courses of the same regimen was given.

Radiotherapy was administered after the completion of chemotherapy, a total dose of 50 Gy in 2.0 Gy daily fractions was delivered to the chest wall and the ipsilateral supraclavicular fossa.

Hormonal therapy was administered according to the hormonal status of the tumour (tamoxifen 20 mg po daily for 5 years) in ER and or PR positive tumours starting after the completion of radiotherapy (patients were followed up 3 monthly during this period).

Patients with progressive disease (PD) at any phase of the protocol therapy were withdrawn and offered alternative treatment accordingly; if they had received at least two preoperative cycles they were included in the toxicity and response evaluations as well as in the survival analysis.

## Dose modification for adverse events

Dosage adjustments were made before each treatment based on blood counts, renal and liver function tests, and other toxicities. Once a dose of chemotherapy was reduced, it was not re-escalated. However, every effort was made to maintain the planned dose intensity and frequency.

## Assessment of Response to Neo-adjuvant Chemotherapy

### Clinical Response

The evaluation of clinical response after primary chemotherapy was performed using the classic World Health Organisation criteria, with physical examination, mammography, and ultrasonography. Moreover, for IBC, apart from the standard criteria, response was additionally defined as the remission or disappearance of inflammatory clinical signs [9].

### Pathological response

Pathological response in the surgically resected specimen was based on exhaustive microscopic examination of multiple sections from the breast and axillary lymph nodes according to the Miller and Payne scoring system [10].

### Ki67 staining procedure

Cryostat sections (5 μm) were cut, mounted on slides coated with tissue adhesive, fixed in acetone (−10 to −25°C) and air-dried. Sections were incubated at room temperature with normal goat serum (diluted 1 in 10 with 10 mm phosphate buffered saline (PBS; pH 7.2–7.4) for 15 min, excess serum was removed and the slides were incubated for a further 45 min with the mouse monoclonal MIB1 which detects the Ki67 antigen (Dakopatts, Denmark). The slides were then rinsed in PBS and reincubated for 30 min with a goat-antimouse bridging antibody (Sigma, UK) containing normal human serum (diluted 1 in 50 with PBS), followed, after washing (2x PBS), with a mouse peroxidase antiperoxidase complex (PAP; diluted 1 in 250 with PBS; Dakopatts, Denmark) for 30 min. A chromogen substrate solution containing hydrogen peroxide (0.06% v/v) and diaminobenzidine 4 HC1 (DAB; 0.05% w/v) was added to each specimen for 5 min. The reaction of peroxidase in the PAP complex with hydrogen peroxide converts the DAB to a reddish brown product. Sections were immersed in distilled water before counterstaining with Harris’s haematoxylin (1% v/v) for 6 min. The slides were then rinsed in tap water for 5 min, dehydrated in alcohol, cleared in xylene and mounted under cover slips in dibutylpthalate xylene solution. All specimen evaluation was performed on an Olympus microscope (BH-2) using an ocular magnification of x40 with an eye piece grid (Graticules Ltd, UK). Ten to 20 fields per tumour were examined depending on its cellularity (minimum 1,000 tumour cells). Control slides (minus primary antibody) were assessed for non-specific binding before assessing the percentage of tumour cell binding the Ki67 antibody. Tumours were classified as positive where greater than 5% of tumour cells expressed detectable quantities of Ki67. This value was selected since it represented a figure which is in excess of that determined for normal and benign breast tissue. Additionally, tumours were classified as having high score if at least 20% of their cells express Ki67 by MIB1 immunostaining. This value was chosen because it selected for the highest quadrile of Ki67 values, whereas tumours with 5–19% of their cells expressing Ki67 immunostaining were classified as having low score. Areas of normal and benign breast were excluded from the final assessment.

### Toxicity Criteria

Toxicity criteria used in this study were those of The NCI Common Terminology Criteria for Adverse Events v3.0.

### Statistical methods

SPSS package (version 12.0) was used for data analysis. Mean and standard deviation were reported to describe quantitative data. The Chi-square and Fischer exact tests were used to evaluate the differences in the distribution of the variables. The Kaplan–Meier method was used to estimate the overall and progression free survival and the Log rank test to evaluate differences in survival among groups.

## Results

A total of 42 patients, aged between 28 and 68 (mean age 47.28 years ± 9.08), of which 21 cases were stage III B (T4d, N0-2 M0) IBC and 21 were stage III B (T4a-c, N0-2, M0) LABC, were included and followed up in this study during the period from November 2007 until February 2010 with a median follow-up period of 22.5 months (5–26).

### Patients’ Characteristics

Table 1 summarises patients’ characteristics with regard to age, menopausal status, performance status, hormonal receptor status and Her2neu expression.

### Comparison between positive DLI in IBC and LABC

In patients with IBC 80% of examined specimens showed positive dermal lymphatic invasion as compared with 16.7% of LABC specimens (P-value ≤ 0.0001).

### Comparison between high score MIB 1 in IBC and LABC

There was a significant difference in the no. of specimens with a high MIB-1 score: 81.3% in patients with IBC as compared with only 43.8% in patients with LABC (P-value = 0.028).

## Toxicity

A total of 164 neo-adjuvant cycles were administered, 82 cycles in each arm. Toxicity of the preoperative phase was, as expected, moderate and manageable in both arms. Haematological toxicity with grade III or IV leucopenia/neutropaenia on day 1 occurred in 23.8% of the patients with IBC and 9.5% of the patients with LABC. Only 4.7 % of IBC cases had febrile neutropaenia, none of them required hospitalisation, whereas none of patients with LABC had febrile neutropaenia. Grade III or IV anaemia occurred in 4.7% of patients with IBC and LABC. No patient required transfusion. No septic or toxic death occurred. Non-haematological toxicities were in general within an acceptable range. Grade III or IV nausea occurred in 23.8% of patients with IBC and LABC. Grade III or IV vomiting occurred in 28.5% of patients with IBC similar to that encountered in patients with LABC. Grade III or IV irregular menses occurred in 85.7% of patients with IBC as compared with 80.9% of patients with LABC. Regarding hepatic toxicity, grade III or IV elevated transaminases occurred in 9.5% of IBC cases and 4.7% of patients with LABC. Grade III or IV hyperbilirubinaemia was observed in 4.7% of patients with LABC. Despite the relatively high cumulative epirubicin dose (600 mg/m2), no significant short- or long-term cardiac toxicity was detected during the median follow-up period. All toxicity results are summarised in table 2.

### Treatment outcome

Seventeen patients of the IBC group underwent modified radical mastectomy compared with 21 patients of LABC group. In the IBC group at the end of a median follow-up of 14.2 (5–25) months, 13 (61.9%) patients are alive and 10 (47.6%) are free of progression whereas in the LABC group after a median follow-up of 22.8 (13–26) months 20 patients are alive and 14 (66.7%) are free of progression.

## Response

### Clinical response to neo-adjuvant chemotherapy

There was a trend towards worse objective clinical response to neo-adjuvant chemotherapy in the IBC arm as compared with the LABC arm. Objective clinical response (complete response (CR) +partial response) to neo-adjuvant chemotherapy was 57.2% (4.8% CR) in the IBC arm as compared with 81% (9.5% CR) in LABC (P-value = 0.09).

### Pathological response to neo-adjuvant chemotherapy

Overall pathological response (complete pathological response, pCR +partial pathological response, pPR) was 35.3% in IBC arm as compared with 40% in LABC arm (P-value = 0.618). All pathological responses in both arms were partial (Miller, Payne G3, G4), no pathological CR (Miller, Payne G5) was achieved.

## Survival

### Progression free survival (PFS)

**Figure 1:**
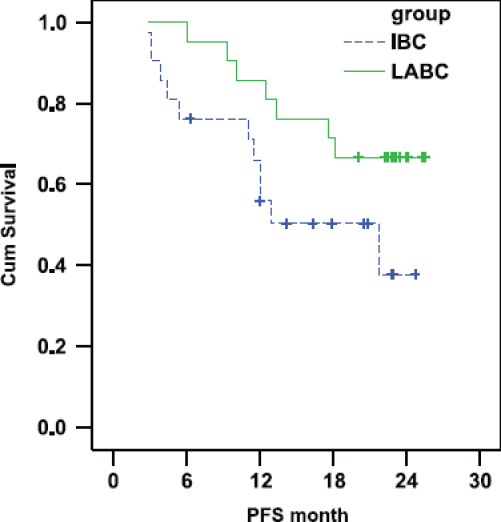
Comparison of PFS in IBC and LABC.

There was a trend towards shorter progression free survival in the IBC arm as compared with the LABC arm. In IBC the PFS was 55.87% and 37.71% at 1 and 2 years, respectively (median PFS: 21.7 months) whereas in LABC the PFS was 85.71% and 66.67% at 1 and 2 years, respectively (median PFS was not reached at the time of statistical analysis (P-value = 0.072) Figure 1).

### Overall survival (OS)

**Figure 2:**
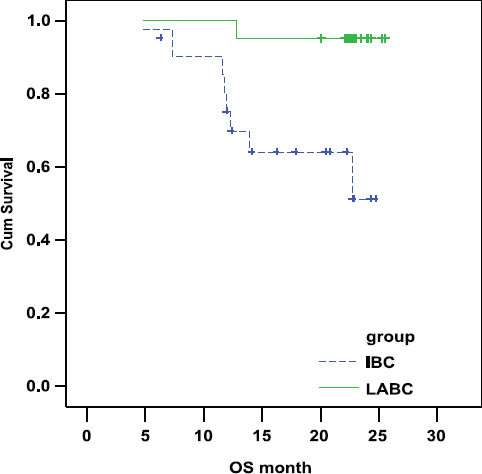
Comparison of OS in IBC and LABC.

There was a statistically highly significant shorter OS in IBC arm as compared with LABC arm. In IBC the OS was 69.28% and 51.20% at 1 and 2 years, respectively. In LABC the OS was 95.24% and 95.24% at 1 and 2 years, respectively (P-value = 0.004; Figure 2).

## Factors predicting survival

### Pathological response

**Figure 3:**
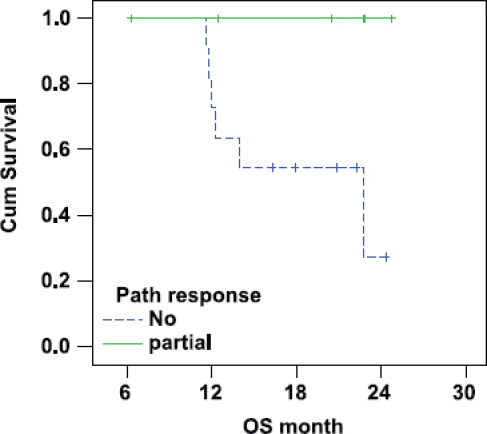
Correlation between pathological response and OS in IBC.

There was a statistically significant lower overall survival in patients with IBC who did not show pathological response (median OS 23 months) as compared with responding patients (median OS was not reached as all patients were alive at the time of statistical analysis (P-value = 0.045; Figure 3).

## Discussion

Inflammatory breast cancer (IBC) is the most aggressive form of primary breast carcinoma and is associated with peculiar clinical and biological features and with a dismal outcome despite multi-modality treatment approaches [1]. The incidence of IBC has been reported to be 0.7 cases per 100 000 person-years by the National Cancer Institute’s Surveillance, Epidemiology, and End Results Program [2].

Studies comparing outcomes in patients with locally advanced breast cancer associated with clinical features of IBC versus those in patients with locally advanced breast cancer without features of IBC demonstrated better outcomes for the latter and a peculiar pattern of recurrence for IBC [2 11–17].

The management of IBC has evolved in the last three decades, resulting in improvements in outcome for this aggressive form of breast cancer [1]. Recent analysis indicates that much of this improvement is related solely to the appropriate use of a multi-disciplinary treatment approach and the introduction of effective chemotherapy [11].

Despite combined multi-modality treatment comprising chemotherapy, surgery and radiation, the prognosis of IBC is poor; the 10-year disease-free survival rate is 20%–25% [1,5,9]. These data clearly indicate that current treatment modalities are inadequate and that a better understanding of the biological features of the disease is necessary if more effective interventions are to be developed.

Although several retrospective analyses have demonstrated that IBC shows peculiar and more aggressive features than locally advanced non-IBC breast cancer, there is still no IBC-specific treatment that could significantly improve the prognosis for these patients [12,18].

For this purpose and to classify IBC as a separate entity, the present study was conducted at the National Cancer Institute (NCI), Cairo University in the period between November 2007 and February 2010. The molecular phenotype including MIB-1 staining, ER, PR, Her2 neuexpression were compared in IBC and stage matched non-IBC. Outcomes of neo-adjuvant chemotherapy including clinical and pathological response were also evaluated.

The study also revealed a statistically significant difference between a high MIB-1 score in the IBC (81.3%) arm than in the LABC arm (43.8%) raising the possibility of using MIB-1 high score as a potential molecular marker for IBC. In a study by Van Golen *et al* IBC had statistically higher Ki67 as compared with non-IBC stage III breast cancer patients (93% versus 11%; P < 0.001 [10]).

We also noted that 42.1% of IBC cases were ER negative as compared with only 19% of LABC cases. In a similar study by Merajver *et al*, the authors also found that IBC is more often oestrogen receptor negative compared with non-IBC; up to two-thirds of the IBC tumours lacked oestrogen receptor expression [16]. In another report 17.9% of IBC cases were oestrogen receptor positive versus 80% of non-IBC cases when analysed by tissue microarray [13]. Response to chemotherapy is one of the most important predictors of outcome. This study showed a trend towards a worse objective clinical response in the IBC arm confirming the more aggressive biological nature of IBC compared with LABC. This study showed no significant difference between pathological responses in both arms, which may be due to the use of conventional chemotherapy, a shorter period of neo-adjuvant chemotherapy and a small sample size making the subgroup analysis inconclusive. This study showed also a statistically significant correlation between pathological response and overall survival in IBC and this is in agreement with the data reported in the literature.

There are few studies on predictive factors for pathological response in IBC. This is probably due to the rarity of IBC and the small patient population. Pathologic complete response (pCR) in breast and axilla to induction chemotherapy has been identified as the strongest prognostic factor for locally advanced breast cancer, including IBC [2,18]. As IBC is a rare disease, patients with IBC were usually treated with the same modalities as, and included in clinical trials designed for, patients with non-inflammatory locally advanced breast cancer. In a review of the data from five studies at the MD Anderson Cancer centre during the period from 1974 to 2001 178 patients were treated as part of four consecutive multi-modality protocols with anthracycline-based regimens. The overall response rate for all four groups combined was 72%, including a 12% clinical CR rate [17,19]. The 2-year OS rate was 71%. The median survival was 37 months for patients on all four protocols combined (38, 38, 64, and 34+ months, respectively). The estimated DFS rates for all 178 patients at 5, 10, and 15 years were 32%, 28%, and 28%, respectively. Patients who experienced a CR or PR after induction chemotherapy had an estimated 15-year DFS rate of 44% and 31%, respectively, and a 15-year OS rate of 51% and 31%, respectively. Those patients who experienced an MR or stable disease (SD) had an estimated 15-year DFS and OS rate of 7%, confirming the prognostic significance of response to induction chemotherapy. A subsequent pilot study was initiated to test the feasibility of using the sequence of FAC plus weekly high-dose paclitaxel as induction chemotherapy. A preliminary analysis of the data revealed that, of the 18 evaluable patients, 7 (31%) experienced a clinical CR and 11 (61%) experienced less than a CR (defined as clinical and radiological persistence of disease). Thirteen patients underwent mastectomy; 6 (46%) of those experienced pathological CR. These data are encouraging, but they are from a small pilot study (number of patients = 20), which limits their use when developing standard-of-care recommendations for patients with IBC [20]. Kuerer *et al* reported a series of 372 patients with locally advanced breast cancer who were all treated with neo-adjuvant anthracycline-based chemotherapy. A total of 43 (12%) patients achieved a pathologic CR, and those patients had a significantly better survival rate [7]. Although the survival time of responders was better than that of non-responders, 13% of the complete responders had relapsed or died by 5 years. Our study revealed a highly significant lower overall survival in IBC arm compared with LABC and also showed a trend towards shorter progression free survival in IBC arm and this is coinciding with the data reported in the literature. Because of the relative infrequency of IBC, no phase III trials have been reported or performed, so all available knowledge is derived from single-arm clinical trials and retrospective chart reviews. Low *et al* [14] reported a long-term follow-up study of 106 patients with locally advanced disease. Those authors retrospectively analysed the outcome of combination chemotherapy in patients with IBC compared with patients with stage III non-IBC. The 10-year OS rates for patients with non-IBC and those with IBC were 44.8% and 26.7%, respectively (p = 0.031). A review of the SEER data that compared IBC with non-IBC clearly showed that IBC had a statistically significantly (p = 0.0001) lower overall survival (OS) rate [2]. In a review of the University of Texas M.D. Anderson Cancer Centre’s experience treating 635 patients with locally advanced breast cancer, including IBC with a median follow-up of 90 months (unpublished data) the median progression free survival (PFS) and OS rates were lower in the group with IBC (214 patients) than in the group with stage III non-IBC (421 patients). Median PFS times were 24 months for patients with IBC (95% confidence interval [CI], 19–29) and 35 months for patients with non-IBC (95% CI, 25–45). Likewise, the median OS times were 42 months for patients with IBC (95% CI, 35–49) and 60 months (95% CI, 47–73) for patients with non-IBC. The data from our study and the above mentioned studies show that IBC is a clinically aggressive disease with an overall worse prognosis than non-IBC. These findings suggest that the underlying molecular determinants of the IBC phenotype will require more investigation so that we can design more effective targeted treatments.

## Conclusions

This study clearly showed that the outcome of inflammatory breast cancer is worse than that of non-inflammatory locally advanced breast cancer and hence the current standard treatment for inflammatory breast cancer, which is more or less similar to standard treatment for locally advanced breast cancer, should be changed to fit the peculiarities of IBC.

We suggest that a quantitative MIB-1 score could be a potential molecular marker for inflammatory breast cancer.

This study generated additional support for the concept that novel treatments based on the biological characteristics of IBC are required if the prognosis for patients with IBC is to improve. This needs to be investigated in future clinical trials.

## Figures and Tables

**Table 1:**
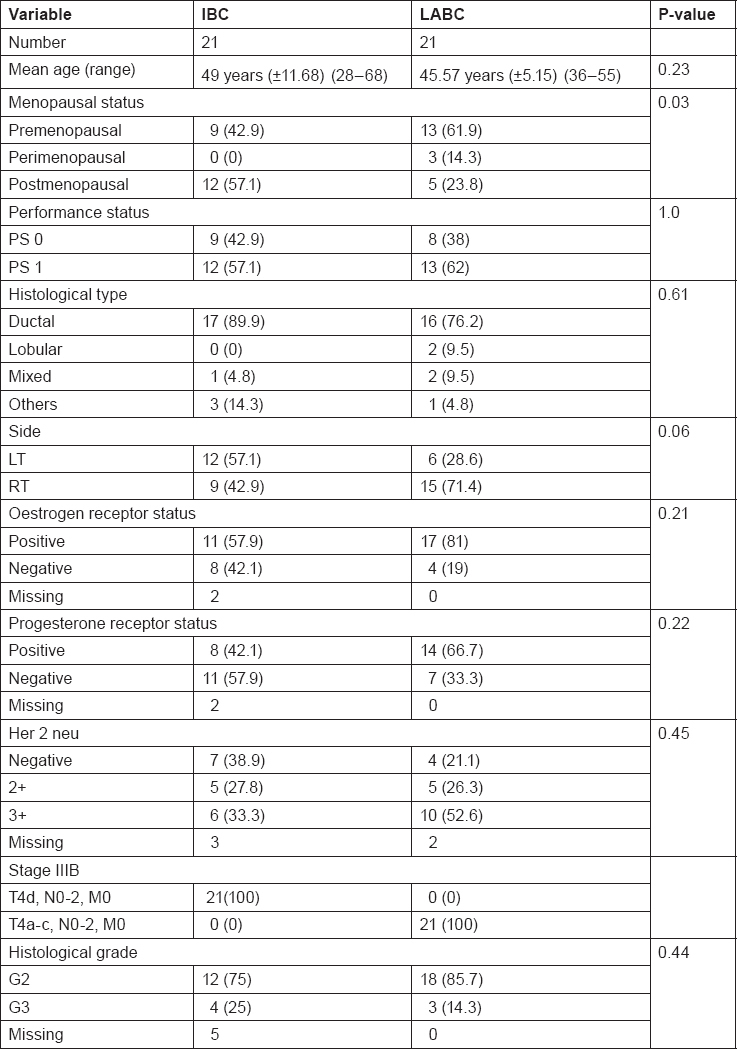
Patients characteristics

**Table 2:**
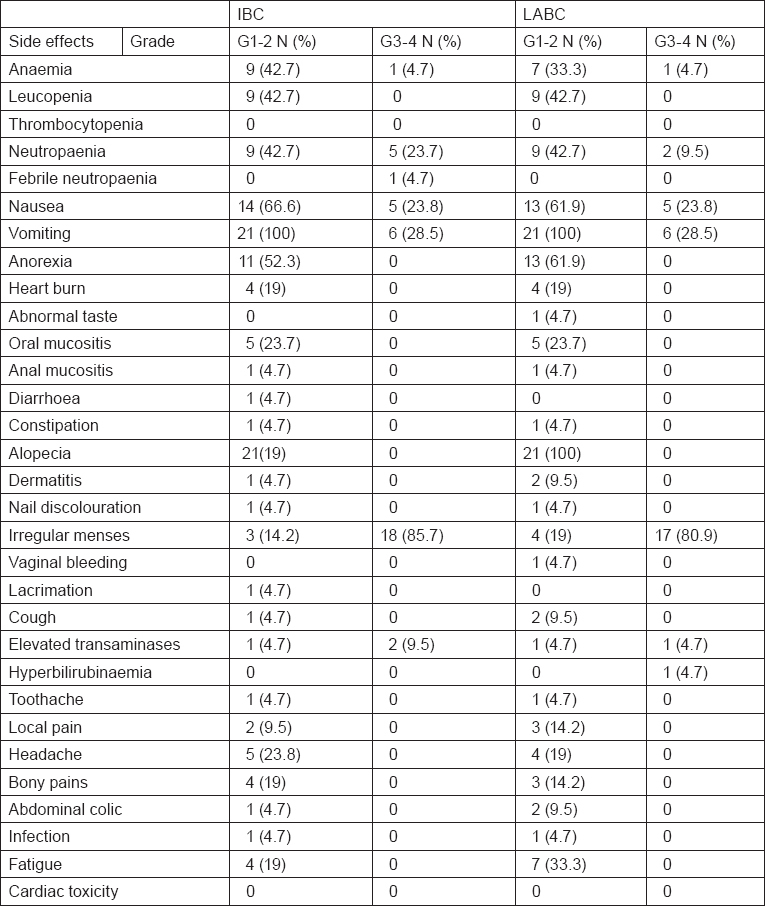
Toxicity of 5-fluorouracil, Epirubicin, and Cyclophosphamide (FEC) chemotherapy in IBC and LABC cases (n=42)
